# The role of stromal cancer-associated fibroblasts in pancreatic cancer

**DOI:** 10.1186/s13045-017-0448-5

**Published:** 2017-03-28

**Authors:** Dagny von Ahrens, Tushar D. Bhagat, Deepak Nagrath, Anirban Maitra, Amit Verma

**Affiliations:** 1 Albert Einstein College of Medicine, 1300 Morris Park Ave, Bronx, NY 10461 USA; 2 Department of Biomedical Engineering, University of Michigan, Ann Arbor, MI 48109 USA; 3 The University of Texas MD Anderson Cancer Center, 1515 Holcombe Blvd., Houston, TX 77030 USA

**Keywords:** Pancreas, Adenocarcinoma, Stroma, Tumor microenvironment, Cancer-associated fibroblast

## Abstract

Pancreatic ductal adenocarcinoma (PDAC) is a lethal cancer generally refractory to conventional treatments. Cancer-associated fibroblasts (CAFs) are cellular components of the desmoplastic stroma characteristic to the tumor that contributes to this treatment resistance. Various markers for CAFs have been explored including palladin and CD146 that have prognostic and functional roles in the pathobiology of PDAC. Mechanisms of CAF-tumor cell interaction have been described including exosomal transfer and paracrine signaling mediated by cytokines such as GM-CSF and IL-6. The role of downstream signaling pathways including JAK/STAT, mTOR, sonic hedge hog (SHH), and NFkB have also been shown to play an important function in PDAC-CAF cross talk. The role of autophagy and other metabolic effects on each cell type within the tumor have also been proposed to play roles in facilitating CAF secretory function and enhancing tumor growth in a low-glucose microenvironment. Targeting the stroma has gained interest with multiple preclinical and clinical trials targeting SHH, JAK2, and methods of either exploiting the secretory capability of CAFs to enhance drug delivery or inhibiting it to prevent its influence on cancer cell chemoresistance. This review summarizes the most recent progress made in understanding stromal formation; its contribution to tumor proliferation, invasion, and metastasis; its role in chemoresistance; and potential therapeutic strategies on the horizon.

## Background

Progress in the treatment of pancreatic adenocarcinoma (PDAC) remains elusive despite substantial time and resources invested in the attempt to improve the dismal prognosis. The American Cancer Society estimates that about 53,070 people will be diagnosed with and about 41,780 will die of pancreas cancer in 2016 with the most recent SEER database reporting a 7.7% five-year survival rate from 2006 to 2012. http://www.cancer.org/cancer/pancreaticcancer/detailedguide/pancreatic-cancer-key-statistics https://seer.cancer.gov/statfacts/html/pancreas.html.

The characteristic desmoplastic stromal response contributes to the challenge in treatment, as it has been shown in multiple studies to promote tumor progression, invasion, metastasis, and chemoresistance [1–6].

**Fig. 1:**
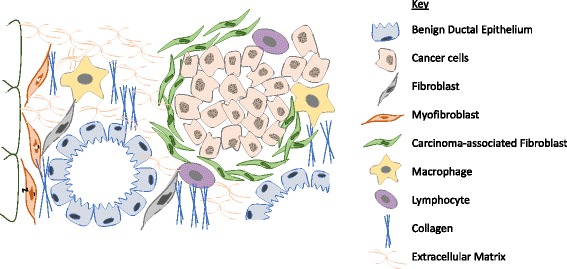
Pancreatic ductal adenocarcinoma with desmoplasia. The PDAC tumor microenvironment is comprised of cellular and acellular components including CAFs, immune cells, and extracellular matrix

The stroma is comprised of cellular components, predominantly cancer-associated fibroblasts (CAFs), immune cells, and a rich extracellular matrix (ECM), all of which interact closely with the tumor cells and offer potential therapeutic targets (Fig. 1) [7–9]. It is encountered as “stickiness” during surgery presenting technical challenges for achieving negative resection margins, which is currently the only chance of cure [10]. Only 20% of patients present at an operative stage in their disease, making effective chemotherapy crucial in the PDAC treatment armamentarium https://www.cancer.gov/types/pancreatic/hp/pancreatic-treatment-pdq. Gemcitabine, the historic gold standard, has only a 23.8% clinical response rate and 6.6-month overall survival (OS) with some improvement after the more recent addition of nanoparticle albumin-bound (nab)-paclitaxel to 8.7 months [11–13]. The most efficacious treatment, a three-drug regimen folinic acid (leucovorin) 5-fluorouracil, irinotecan, oxalaplatin (FOLFIRINOX), often cannot be tolerated due to high toxicity and patients’ poor performance status [14, 15].

Treatment shortcomings are increasingly attributed to the stroma inciting chemoresistance in the PDAC cells, as well as decreasing microvascularity and, therefore, drug delivery [3, 5, 6]. Contrary to these tumor-protective principles, two groups, Ozdemir et al. and Rhim et al., both found that outright depletion of stroma actually led to more aggressive tumors with decreased OS, emphasizing the complexity of the stromal-tumor interaction [16, 17]. We aim to provide an update on the progress made in understanding the CAF stromal component with an overview of stromal formation; characterization of its role in tumor progression, invasion, and metastasis; and the mechanisms of stromal influence on PDAC chemoresistance. Potential therapeutic strategies can be derived from much of this new knowledge and a number of clinical trials are in development or currently underway.

## Formation and characterization of PDAC-associated stroma

**Fig. 2:**
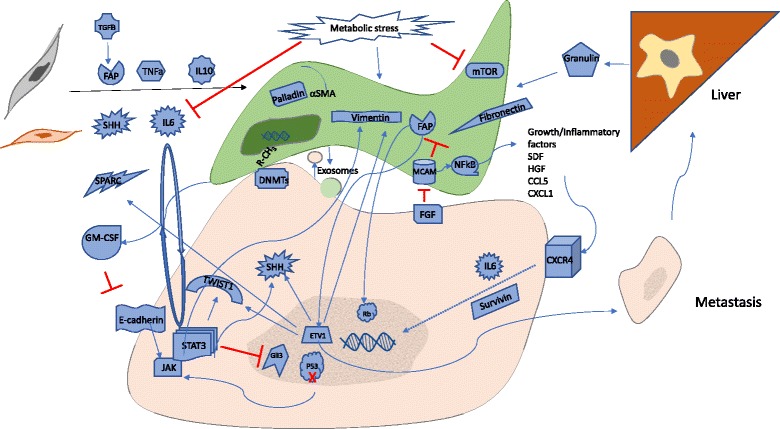
Stromal activation and tumor-stromal interaction. Multiple complex pathways of CAF activation have been found. Once activated, CAFs closely interact with tumor cells through various mechanisms leading to tumor growth, invasion, and metastasis

CAFs develop from bone marrow-derived mesenchymal stem cells (MSCs), pancreatic stellate cells (PSCs), and quiescent resident fibroblasts through multiple pathways of activation including epithelial-mesenchymal transition (EMT) (Fig. 2). Previously described pathways of CAF activation include sonic hedgehog (SHH), TGF-β, TNF-α, interleukins 1, 6, and 10 [18–21]. CAFs are formed from MSCs recruited from the bone marrow that are aided by growth factors and cytokines such as CCL2, hepatocyte growth factor, and fibroblast growth factor (FGF) [22–24]. Once activated, CAFs of all origins take on a largely secretory and contractile function. Histologic characterization of PDAC stroma continues to be ongoing. In 2008, Erkan et al. described four collagen deposition patterns and determined that up to 80% of the tumor volume is comprised of stroma [25]. Lakiotaki et al. most recently described CAFs in human pancreatic tumors as densely arranged around all carcinomatous structures in a complete or incomplete perineoplastic ring. CAFs were scant, however, around all benign tissue and ducts [26].

The group also newly described vascular elastotic changes seen most prominently within the neoplastic tissue and less prevalent at the tumor periphery. Interestingly, no elastic fibers were seen around carcinomatous structures or within the stroma. Native fibroblasts, smooth muscle cells, and endothelial cells typically produce elastin, and native fibroblasts are localized around the duct and adventitia before activation into CAFs. Their findings indicate that these CAFs do not seem to be involved in the elastic fiber deposition given the differing location of the cells and fibers, although the possibility of degradation by matrix metalloproteinases (MMPs) should be considered [26].

Investigations into other carcinomatous tumors allude to novel mechanisms of stromal activation including exosomal transformation of mRNA from cancer cells [27], as well as paracrine and autocrine activation of cancer-promoting pathways such as CXCR4-SDF1A [27, 28]. Epigenetic mechanisms including DNA methylation have also been found to regulate genes involved in formation of ECM [29]. Kohi et al. most recently demonstrated that production of hyaluronan (HA)—an abundant ECM component in PDAC and a marker of poor prognosis—is regulated by DNA methylation as demonstrated by the upregulation of hyaluronan synthase with a DNA methylating agent and confirmation by knockdown of DNA methyltransferase 1 [29]. Another group’s findings suggest that PDAC cells reprogram stromal cells through DNA methylation of genes such as SOCS1 through direct cell-to-cell contact rather than paracrine signaling [30].

## Cellular markers for CAFs

The heterogeneity demonstrated within CAF populations necessitates reliable markers. Some have been established, but a true, unique marker is yet to be discovered. Native fibroblasts stain for vimentin and, once activated, stain positive for alpha-smooth muscle actin (αSMA) [31]. Other CAF markers previously reported include stromal cell-derived factor-1α (SDF1A), fibroblast activation protein (FAP), and fibroblast-specific protein-1 (FSP-1) [28, 32]. More recent studies, such as that by Cannon et al., found that palladin, an actin-binding protein highly expressed in cancers, actually precedes αSMA expression in cells undergoing activation to CAFs. The group further elucidated the role of palladin isoform 4 in CAF activation including its location in the cytoplasm and nucleus, and its role in expression of genes associated with collagen formation and MMP activation pathways demonstrated by shRNA knockdown [31].

## Molecular pathways regulating stromal functionality

Various cellular pathways have been implicated in regulating CAF functions. Recently, one of four key mutations in PDAC tumorigenesis, loss of TP53, has been shown to influence stromal formation as well. A recent study demonstrated that the TP53 mutation is correlated with STAT3 phosphorylation upon IL-6 exposure and persists in a feed-forward autocrine loop. They further showed that persistent STAT3 activation upregulates SHH and suppresses GLI3, a transcription factor known to suppress stromal formation. They confirmed these findings in vivo, demonstrating that a JAK2 inhibitor depleted the stroma and resulted in densely packed ductal tumor cells with decreased PSC activation, as well as diminished and altered collagen structures [33].

Negative regulation of stromal formation was observed to occur by CD146—also known as melanoma-specific cell adhesion molecule (MCAM)—signaling. CD146 knockdown increased the viability of CAFs, as well as the expression of fibroblast activation protein (FAP) and fibronectin, additional known markers of CAF activation. The reciprocal findings were demonstrated with CD146 overexpression further confirming their findings [34].

During activation, CAFs undergo metabolic stress, which activates autophagy, by inducing protein kinase B (AKT) and inhibiting mTOR signaling pathways to meet the increased energy demands of a secretory cell. Using the mitochondrial uncoupling agent, rottlerin, Su et al. demonstrated that metabolic stress altered gene expression of ECM proteins and upregulated CHOP, a pro-apoptotic transcription factor modulating cell fate [35]. Additionally, the pro-tumorigenic cytokine IL-6 was significantly downregulated by metabolic stress but IL-4, an immune modulator, was significantly upregulated.

## Role of stromal cells in cancer proliferation, progression, and invasion

Once activated, the PDAC stroma plays a dynamic part in tumor cell proliferation and invasion, the mechanisms of which are gradually being elucidated (Fig. 2) [1, 2, 36]. In addition to autocrine and paracrine influences of CAFs on PDAC tumor cells, translocation of metabolic substrates have been shown to transfer from CAFs to tumor cells via exosomes. Zhao et al. demonstrated by isotope tracing that exosomes from CAFs containing lactate, acetate, amino acids, lipids, and tricarboxylic acid (TCA) cycle intermediates actually reprogrammed cancer cells to inhibit mitochondrial oxidative phosphorylation and upregulate glycolysis and glutamine-dependent reductive carboxylation likely via miRNA and substrate transfer [37]. Another metabolic study demonstrated that CAF autophagy, stimulated by tumor cells, causes alanine secretion which actually outcompetes glutamine and, in turn, provides fuel for those tumor cells in its low-glucose microenvironment [38].

It has been reported that the transcription factor ETV1 plays an important role in orthotopic xenograft models of PDAC in mice. Heeg et al. found that ETV1 overexpression doubled tumor volume by stromal expansion, altered the stromal morphology, increased invasive capacity, and upregulated EMT and MMP regulators including SLUG, SNAIL, TWIST, vimentin, ZEB1, ZEB2, and MMP9. They also identified secreted protein acidic cysteine-rich (SPARC) and hyaluronan synthetase 2 (HAS2) as important separate downstream targets, both known modulators of stromal expansion [39]. In fact, SPARC knockdown completely abrogated the pro-tumorigenic effects of ETV1 overexpression solidifying this pathway link.

Another key player in CAF-tumor cell cross talk is FAP, which has been shown to play a critical role in ECM formation, angiogenesis, cell motility, immune suppression, and ultimately clinical outcome [40]. Kawase et al. confirmed the role of FAP in cancer cell invasion and EMT and additionally showed its role in the activation of cell cycle progression by phosphorylation of Rb protein in PDAC cells. They also demonstrated that TGF-β, which has been linked to induction of EMT, induces FAP expression [41].

The aforementioned negative regulator of stromal formation, CD146 or MCAM, was also found to have a role in tumor progression and invasion. Zheng et al. identified a CD146-positive subpopulation of CAFs in patient samples and found that CD146-negative patients were more likely to have higher tumor grade, clinical stage, and likelihood of residual tumor post-operatively. CD146-positive patients also had increased disease-free time post-operatively with more than double median survival. Knockdown of CD146 was found to enhance tumor cell migration and invasion and most notably upregulated growth factors and pro-inflammatory genes including SDF1A, CXCL1, CCL5, hepatocyte growth factor (HGF), and COX2, likely via NFkB suppression. Interestingly, cancer cells were identified as a potential downregulator of CD146 after identifying FGF—produced by PDAC cells—as an inhibitor of CD146 and inducer of NFkB [34].

This heterogeneity within stromal cells is becoming more apparent. Waghray et al. identified a subpopulation of CAFs designated as cancer-associated mesenchymal stem cells (CA-MSCs) and demonstrated their role in invasion as mediated by granulocyte-macrophage colony-stimulating factor (GM-CSF) [42]. Their findings mirrored those of Heeg et al. in demonstrating larger tumors with the addition of CA-MSCs for in vivo models, but they also demonstrated increased proliferation of cancer cells themselves with increased Ki67 expression as well as stromal expansion [39]. The group further demonstrated a mechanism through which GM-CSF production by CA-MSCs induces invasion by downregulation of E-cadherin and upregulation of TWIST1 and vimentin via the JAK2/STAT3 pathway, suggesting a link to results obtained by Wormann et al. [33].

## Role of CAFs in regulating metastasis

Many of the mechanisms involved in stromal influence on tumor progression and invasion also exhibit influence on metastasis. Unique and dynamic features of stromal components of the metastatic lesions themselves are also beginning to be described [33, 39, 43]. Aiello et al. characterized stroma within metastatic lesions in an autochthonous model of PDAC. They showed that myofibroblasts appear when metastases are as small as 6–7 cells and that cell populations within these lesions become more epithelial during growth. They found that stromal volume, including cellular and extracellular components, eventually reaches levels similar to primary lesions [43]. Heeg et al. found that overexpression of transcription factor ETV1 drastically increased the incidence and volume of micro- and macrometastasis in mouse models. Those metastatic lesions were also observed to have a marked stromal expansion themselves. SPARC1 knockdown in mice had similar metastatic rates to controls demonstrating this protein’s role in the ETV1-induced pathway [39].

Nielson et al. elegantly demonstrated that the regulation of stroma within PDAC liver metastases is unique and dependent on immune interactions, which may actually precede cancer cell metastasis. The group injected mice intrasplenically with KPC, a murine PDAC cell line, and found that immune cells—initially inflammatory monocytes followed by metastasis-associated macrophages (MAMs)—accumulated in the liver. They did not detect αSMA-marked stromal expansion in these injected mice; however, expansion was seen in the established experimental and spontaneous mouse tumor metastases. They further demonstrated that these MAMs were bone marrow-derived rather than native Kupffer cells. In contrast, the metastasis-associated fibroblasts were found to be of local origin, presumably hepatic stellate cells. Similar indications that the tumor microenvironment facilitates homing and invasion have been described in colon cancer and other tumors [44, 45].

This same group showed through abolition of macrophage trafficking that the incidence of metastasis and fibroblast activation were decreased after intrasplenic KPC injection. Chemical ablation of MAMs in these mice at a time point after metastatic seeding had occurred to also decreased accumulation of activated myofibroblasts and reduced the size of the area covered by metastatic cells, though it did not significantly reduce the metastatic frequency. Macrophage-conditioned media was demonstrated to strongly activate quiescent fibroblasts, and by secrotome analysis, granulin was established as the main effector. Interestingly, granulin was expressed in the bone marrow-derived macrophages of the liver metastases but not in those macrophages found at the primary tumor site. They also showed that periostin, a paracrine stimulator of tumor cells, was also dependent on granulin in its pro-tumerigenic effects, again as evidence of the cross talk within the PDAC milieu [4].

## Role of stromal cells in chemoresistance

The stroma has been shown to facilitate chemoresistance through physical barrier methods as well as paracrine cross talk and transformation of tumor cells [3, 5, 6]. Wormann et al. demonstrated that gemcitabine, when administered with a JAK2 inhibitor, markedly decreased tumor growth and increased overall survival [33]. Another modulator of chemoresistance appears to be the SDF1A/CXCR4 axis. The known inducer of proliferation, migration, and invasion was also found to facilitate chemoresistance through FAK/AKT with autocrine activation of IL-6 [46]. SDF1A was expressed in CAFs while CXCR4 was active in PDAC cells, where IL-6 was also induced, again demonstrating the close interplay between stromal and tumor cells. Duluc et al. confirmed the role of IL-6 in tumor-CAF cross talk and also demonstrated the importance of the mTOR/4E-BP1 axis on imparting chemoresistance. The group also found that secreted factors from CAFs including IL-6 induced cancer cell production of survivin, an inhibitor of apoptosis [47].

Disparate results have called into question the physical barrier theory of stroma imparting chemoresistance. Most recently, Aiello et al. demonstrated in mouse models a widespread reduction in metastases with chemotherapy administration both in lesions with minimal and dense stroma. Little is known though about the character and function of stroma within PDAC metastases [43]. Zechner et al. did find that, within primary tumors treated with a combination of metformin and gemcitabine, metformin better inhibited cancer cells near the desmoplastic stroma, whereas gemcitabine inhibited proliferation in cells distant to the stroma [48]. Hesler et al. showed that CAFs serve as sources of CYR61 in co-culture models and induce chemoresistance by downregulating nucleoside transporters that mediate cellular uptake of gemcitabine [49]. Together, these studies indicate that further research is needed to understand the ability of PDAC to develop chemoresistance in order to either exploit or overcome the stromal component of the tumor.

## Therapeutic strategies targeting stromal cells

Many of the studies in this review reveal potential druggable targets, some of which have already been investigated both preclinically and, some, clinically (Table 1). The first stroma-targeting treatments on clinical trial were SHH inhibitors. Though multiple trials continue to be ongoing, a number have failed with striking discrepancy in results from preclinical data.

**Table 1:** PDAC stroma-associated therapeutic targets

Drug	Target	Mechanism	Preclinical results	Clinical results	Author
All-*trans* retinoic acid + gemcitabine	PDAC and stroma	CAF deactivation to quiescence with vitamin A storage capacity	Decrease in transcription factors in PDAC cells Increased markers of stromal quiescence Decrease in EMT	N/A	Carapuca et al. [50]
SPARC overexpression + nab-paclitaxel	PDAC via stroma	Sequestration of drug by SPARC	2.8× gemcitabine concentration within tumor	50% reduction in PDAC cell proliferation Increase in endothelial proliferation	Von Hoff et al. [12]
PEGPH20 + gemcitabine	Stroma	Stromal degradation by hyaluronidase	Significant depletion of hyaluronan 4× increase microvessel lumen diameter Increase in drug delivery to tumor within first 24 h	7.2-month progression-free survival (PFS) and 12-month OS in tumors highly expressing hyaluronan Overall response rate of 25 and 67% in high HA tumors vs. 13% in standard therapy Minimal side effects	Hingorani et al. [52]
Momelotinib, ruxulitinib + nab-paclitaxel or gemcitabine	JAK2	JAK2 inhibition	Depletion of stroma Decreased PSC activation Diminished and altered collagen structures	OS 0.79 and PFS 0.72 hazard ratio (HR) ruxolitinib vs. placebo High inflammation subgroup: OS HR 0.47 vs. placebo Ruxolitinib failed phase III, momelotinib ongoing	Hurwitz et al. [53] Dawkins et al. Koh et al. Wormann et al. [33]
SHH inhibitors	SHH	Decreased stroma expansion	Conflicting results	Conflicting results	Olive et al. [5] Kim et al. [54] Laheru et al. Dejesus-Acosta et al.
Pasireotide	CAFs	Somatostatin analog	Reduced tumor growth Reduced chemoresistance	N/A	Duluc et al. [47]

In another therapeutic approach utilizing all-*trans* retinoic acid (ATRA)—chosen for the known vitamin A storage capability of quiescent PSCs—the study drug, when combined with gemcitabine, reduced cell proliferation and tumor invasion and also enhanced apoptosis of cancer cells. ECM deposition and CAF invasive ability and density were diminished both in vitro and in vivo. PSC deactivation was also indicated by an increase in retinoic acid receptor beta (RARB), which marks quiescence. Their findings also suggested a decrease in signaling related to EMT through downregulation of Wnt signaling in PSCs and reduction of nuclear TWIST1 and ZEB1 transcription factors in PDAC cells with ATRA administration [50].

CAF storage capability can also be harnessed for drug delivery as Bonomi et al. demonstrated. The group primed mesenchymal stroma cells of pancreas and bone marrow origin with 2000 ng/ml of gemcitabine. After co-culturing each with PDAC cells in vitro, they found a 50% reduction in PDAC cell proliferation [51]. Previous work also demonstrated similar efficacy of this drug delivery method for paclitaxel [12]. The drug was described to deplete the stroma as less collagen was seen and neoplastic glands became more densely packed, but the group did not stain for αSMA or vimentin directly. There was increased endothelial staining and a 2.8-fold increase in gemcitabine concentration within the tumor [12]. SPARC, present in the tumor stroma, was postulated to sequester nab-paclitaxel contributing to its efficacy in preliminary clinical trials. An association has been drawn between SPARC and treatment response with an increased overall survival; however, the presence of SPARC generally has been associated with a poorer prognosis [12].

Encouraging results of a phase Ib clinical trial may point to the future of stromal manipulation in PDAC treatment. Pegylated recombinant hyaluronidase (PEGPH20) in combination with gemcitabine was well tolerated with manageable musculoskeletal side effects. Efficacy was analyzed for tumors expressing high levels of hyaluronan (HA) histologically vs. low expression. Progression-free survival (PFS) and OS was 7.2 and 13 months for high HA tumors and 3.5 and 5.7 months for low HA tumors compared to the current standard of care chemotherapy regimens of 8.5 months for nab-paclitaxel plus gemcitabine and 11.1 months for FOLFIRINOX. Preclinical data demonstrate significant depletion of hyaluronan and a four-fold increase in microvessel luminal diameter, thereby increasing drug delivery within 24 h of tumor exposure. Together, the results of this small preliminary clinical trial suggest that PEGPH20 may be most efficacious in tumors expressing high levels of HA [52].

The JAK2 inhibitor momelotinib is currently under investigation as an adjunct to nab-paclitaxel and gemcitabine. Phase II trial data of another JAK2 inhibitor, ruxolitinib, demonstrated significant improvement in OS within a high-inflammation subgroup; however, the phase III trial was recently terminated due to lack of demonstrated efficacy [53].

Duluc et al. effectively used pasireotide, a secretory inhibitor similar to somatostatin to inhibit CAFs’ secretory capabilities, and found that it reduced tumor growth and chemoresistance [51]. Somatostatin analogs have been safely used clinically for decades, providing a promising avenue for clinical exploration much of which has already been undertaken in pancreatic neurendocrine tumors.

## Conclusions

Research into PDAC stroma is rich with potential for improving treatment of this lethal tumor. Many aspects of the desmoplastic response have been reviewed here including mechanisms of stromal formation, influence on tumor progression, invasion, mestastasis, and chemoresistance, but further original research is needed to make a significant impact for patients with pancreatic cancer.

## Abbreviations

ATRA: All-*trans* retinoic acid
CAF: Cancer-associated fibroblast
CA-MSCs: Cancer-associated mesenchymal stem cells
CHOP: CCAAT-enhancer-binding protein homologous protein
ECM: Extracellular matrix
EMT: Epithelial-mesenchymal transition
FAP: Fibroblast activation protein
FGF2: Fibroblast growth factor 2
FOLFIRINOX: Folinic acid (leucovorin) 5-fluorouracil, irinotecan, oxalaplatin
HA: Hyaluronan
HAS2: Hyaluronan synthetase 2
HGF: Hepatocyte growth factor
HR: Hazard ratio
KPC: G12D/+; p53R172H/+; PdxCretg/+ murine PDAC cell line
MAM: Metastasis-associated macrophages
MCAM: Melanoma-specific cell adhesion molecule
MMP: Matrix metalloproteinase
nab-paclitaxel: Nanoparticle albumin-bound paclitaxel
OS: Overall survival
PDAC: Pancreatic ductal adenocarcinoma
PEGPH20: Pegylated recombinant hyaluronidase
PFS: Progression-free survival
PSC: pancreatic stellate cell
RARB: Retinoic acid receptor beta
SDF1A: Stromal cell-derived factor 1 alpha (CXCL12)
SEER: Surveillance Epidemiology and End Results Program
SHH: Sonic hedge hog
SPARC: Secreted protein acidic cysteine-rich
TCA: Tricarboxylic acid cycle
TGF-B: Transforming growth factor beta
TNF-α: Tumor necrosis factor alpha
αSMA: alpha-Smooth muscle actin
